# Early detection of Alzheimer’s disease via multimodal MRI and machine learning

**DOI:** 10.3389/fnagi.2026.1794982

**Published:** 2026-04-13

**Authors:** Jin Yuan, Yingying Liu, Juntao Jin, Siyu Li, Yamin Zhang

**Affiliations:** 1 The First Clinical Medical College, Gansu University of Chinese Medicine, Lanzhou, China; 2 Department of Neurology, Gansu Provincial Hospital, Lanzhou, China

**Keywords:** Alzheimer’s disease, diffusion tensor imaging, early diagnosis, machine learning, resting-state functional MRI

## Abstract

**Objective:**

This study aimed to identify effective biomarkers associated with early-stage Alzheimer’s disease (AD) by integrating multimodal neuroimaging features with machine learning (ML), addressing clinical challenges posed by global population aging.

**Materials and methods:**

Multimodal neuroimaging—including resting-state functional MRI (rs-fMRI), structural MRI (sMRI), and diffusion tensor imaging (DTI)—was combined with ML techniques. A total of 234 subjects [cognitively normal (CN), mild cognitive impairment (MCI), and AD] were selected from the AD Neuroimaging Initiative (ADNI) database. Brain functional, structural, and microstructural features were extracted, and nine ML models, including support vector machine (SVM), random forest (RF), and Naive Bayes, were trained and evaluated across three binary classification tasks: AD-CN, MCI-CN, and AD-MCI.

**Results:**

The SVM model achieved the highest performance for AD-CN (AUC = 0.901) and MCI-CN (AUC = 0.839), while RF performed best for AD-MCI classification (AUC = 0.809). Functional analyses revealed significant abnormalities in key regions, including the anterior cingulate cortex, hippocampus, and middle frontal gyrus in AD patients. Structural analyses confirmed that hippocampal subfield atrophy was strongly associated with cognitive decline. Diffusion metrics, particularly the DTI-ALPS index, reflected microstructural white matter damage effectively.

**Conclusion:**

Integrating multimodal neuroimaging with ML enhances diagnostic accuracy for AD and MCI and identifies potential neuroimaging biomarkers, providing objective evidence to support early clinical intervention.

## Introduction

Alzheimer’s disease (AD) is a prevalent neurodegenerative disorder of the central nervous system, characterised by progressive cognitive decline and behavioural impairment, primarily affecting elderly and pre-elderly populations. Clinically, AD typically manifests as early memory impairment, followed by aphasia, agnosia, visuospatial dysfunction, impaired abstract reasoning, and reduced computational ability (Alzheimer’s Association, 2023). Personality and behavioural changes are also common. As the disease advances, patients gradually lose independence in activities of daily living and ultimately become fully dependent on caregivers (Alzheimer’s Association, 2023). Despite substantial research efforts, the pathogenesis of AD remains incompletely understood, and no curative treatment is currently available. Existing therapies mainly aim to delay disease progression and improve quality of life. According to the Global Burden of Disease study, AD is the fifth leading cause of death worldwide. In China, the number of individuals with AD and other dementias has reached 16.99 million, with prevalence and mortality rates slightly exceeding the global average. With rapid population ageing, AD has become a major public health burden in China.

Early identification of AD, particularly at the stage of mild cognitive impairment (MCI), is essential for timely intervention (Lombardi et al., 2020). Structural magnetic resonance imaging (sMRI) can detect macroscopic brain alterations; however, these changes do not always closely reflect the underlying pathological processes of AD (Beheshtian et al., 2021; Gonneaud et al., 2021). In contrast, resting-state functional MRI (rs-fMRI), which measures spontaneous blood oxygen level–dependent (BOLD) signal fluctuations, enables the assessment of intrinsic brain activity and functional network organisation and is more sensitive to early functional abnormalities, even before overt clinical symptoms appear (Shinn et al., 2023). Diffusion tensor imaging (DTI), which characterises white matter microstructural integrity, has also been shown to be valuable for the early diagnosis of AD and MCI (Greener et al., 2022; Rauschecker et al., 2020; Quintas-Neves et al., 2024). The combination of these multimodal MRI techniques allows a more comprehensive characterisation of AD-related structural and functional brain changes, thereby improving diagnostic accuracy.

In recent years, machine learning (ML) methods have shown substantial potential in the early diagnosis of AD (You et al., 2025; Singh et al., 2024; Jann et al., 2025). By automatically analysing high-dimensional neuroimaging data, ML algorithms can identify subtle pathological patterns that are difficult to detect through conventional visual assessment, providing objective and quantitative diagnostic support. Feature extraction and pattern recognition from sMRI, rs-fMRI, and DTI data enable ML approaches to detect early AD-related alterations and assist clinicians in identifying patients before clear clinical symptoms emerge (Fan et al., 2024). Integrating ML with multimodal neuroimaging has been shown to improve diagnostic performance and support more accurate clinical decision-making (Sheng et al., 2024; Basaia et al., 2019; Lin et al., 2023).

In this retrospective cross-sectional study, we aimed to investigate whether multimodal MRI features could support discrimination among cognitively normal, mild cognitive impairment, and Alzheimer’s disease groups at a single time point. Structural, microstructural, and functional features derived from sMRI, DTI, and rs-fMRI were jointly analysed and evaluated across multiple machine-learning classifiers to assess their potential value for disease-stage classification. In addition, Shapley Additive Explanations (SHAP) analysis was used to improve model interpretability by identifying imaging features that contributed most strongly to model predictions. Rather than inferring causal mechanisms or longitudinal disease progression, the present study focused on the cross-sectional characterization and classification value of multimodal neuroimaging features in the Alzheimer’s disease spectrum.

## Materials and methods

### Study participants

This study was a retrospective cross-sectional analysis based on data obtained from the Alzheimer’s Disease Neuroimaging Initiative (ADNI).^1^ The primary objective was to characterize multimodal MRI features associated with group differences among cognitively normal (CN), mild cognitive impairment (MCI), and Alzheimer’s disease (AD) participants and to evaluate their discriminative value for machine-learning-based classification at a single time point. Only participants with complete sMRI, rs-fMRI, and DTI data acquired within the same study visit were included. Participants with missing modality data, failed quality control, or unusable images after preprocessing were excluded. After quality control and manual screening, 234 participants were enrolled, including 37 patients with AD, 101 individuals with MCI, and 96 CN controls. All data usage complied with the ADNI data-sharing protocol.

### rs-fMRI data preprocessing

Resting-state fMRI preprocessing was conducted using DPARSF (version 4.2)^2^ in MATLAB 2022b. Preprocessing steps included format conversion, removal of the first 10 volumes, slice-timing correction, and head-motion correction (exclusion criteria: >2 mm translation or >2° rotation). Images were spatially normalised and resampled to 3 mm isotropic voxels. Regional homogeneity (ReHo), amplitude of low-frequency fluctuations (ALFF), and fractional ALFF (fALFF) were calculated within the 0.01–0.08 Hz band, followed by Z-score normalisation and 6 mm FWHM spatial smoothing. The resulting ALFF, fALFF, and ReHo maps were subsequently used for group-level statistical analysis and downstream feature extraction for machine-learning modeling.

### sMRI data preprocessing

Structural MRI data were processed using SPM12^3^ and the CAT12 toolbox in MATLAB 2022b for voxel-based morphometry (VBM) analysis. Procedures included tissue segmentation, modulation with volume-preserving nonlinear registration, and smoothing with an 8 mm FWHM Gaussian kernel. Whole-brain grey matter volumes and hippocampal and amygdala subfield volumes were extracted for subsequent analyses.

### DTI data preprocessing

DTI preprocessing was performed using dcm2niix, MRtrix3, and FSL. Steps included denoising, Gibbs-ringing artefact removal, bias-field correction, and eddy-current and head-motion correction using a linear sliding least-squares model (slm = linear). Signal-to-noise ratio maps were generated for quality control. Tensor fitting was conducted to derive fractional anisotropy (FA), mean diffusivity (MD), axial diffusivity (ADax), and radial diffusivity (RD) maps (Luppi et al., 2023).

For spatial normalisation, individual FA images were linearly registered to the JHU-ICBM-FA-1 mm template, followed by nonlinear registration of diffusion tensor components (Dxx, Dyy, Dzz). Diffusion metrics were extracted using spherical regions of interest (ROI; diameter = 5 mm) in the bilateral superior corona radiata and superior longitudinal fasciculus based on the JHU-ICBM-DTI-81 atlas. These measures were used to calculate the DTI–analysis along the perivascular space (DTI-ALPS) index and hippocampal subfield diffusion parameters (Hsu et al., 2023). Diffusion-derived metrics and ALPS-related measures were then included in the multimodal feature set for downstream analyses.

### Site effect harmonisation

To mitigate multisite effects, ComBat^4^ harmonisation was applied across rs-fMRI, sMRI, and DTI features. ComBat models site-related variability as additive and multiplicative effects using empirical Bayes estimation, thereby reducing scanner-related confounding while preserving biological variability. To reduce the risk of information leakage in the machine-learning analyses, ComBat harmonisation was performed using training data only, and the estimated harmonisation parameters were subsequently applied to the corresponding held-out test data.

### Machine learning modelling

The machine-learning analysis in the present study focused primarily on multimodal neuroimaging features. Binary classification tasks (AD vs. MCI, MCI vs. CN, and AD vs. CN) were performed within a multimodal machine-learning framework. For each task, subjects were divided into a training set and an independent test set. The training set was used exclusively for preprocessing, feature selection, model development, and hyperparameter tuning, whereas the held-out test set was reserved only for final performance evaluation.

To reduce the risk of information leakage, feature standardization was performed using parameters estimated from the training data only and then applied to the corresponding test data. Feature selection was conducted using least absolute shrinkage and selection operator (LASSO) regression within the training data only. Given the relatively limited sample size and class imbalance, model performance was interpreted cautiously, and no resampling procedure was applied to the held-out test set. Hyperparameters were optimized by grid search with stratified 10-fold cross-validation implemented within the training set. Nine classifiers were evaluated, including support vector machine (SVM), extreme gradient boosting (XGBoost) (Haque et al., 2021), k-nearest neighbour (KNN), random forest (RF), multilayer perceptron (MLP), logistic regression (LR), Naive Bayes (NB), linear discriminant analysis (LDA), and stochastic gradient descent (SGD) classifiers (Archer et al., 2019). Model performance on the independent test set was assessed using accuracy, precision, recall, and area under the receiver operating characteristic curve (AUC). SHAP analysis was applied to the best-performing model for each classification task to quantify the relative contribution of selected features to model predictions and improve interpretability.

### Statistical analysis

Group differences among CN, MCI, and AD were analysed using a hierarchical statistical framework. Normality and variance homogeneity were assessed using Shapiro–Wilk and Levene’s tests. One-way ANOVA or Kruskal–Wallis H tests were applied as appropriate, followed by Tukey’s HSD *post hoc* comparisons. Imaging variables included rs-fMRI (ALFF, fALFF, ReHo), sMRI-derived measures (grey matter and hippocampal/amygdala subfield volumes), and DTI metrics (FA, MD, ADax, RD, hippocampal subfield parameters, and DTI-ALPS index). Age, sex, and education were included as covariates, with total intracranial volume additionally adjusted for grey matter analyses. Multiple comparisons were corrected using the false discovery rate (FDR; *p <* 0.05). Spearman correlation analyses were conducted to examine associations between imaging metrics and cognitive scores, including MMSE, MoCA, ADAS-Cog, and RAVLT.

## Results

### Demographic and clinical characteristics

Demographics and clinical assessments are summarised in Table 1. Groups differed significantly in age (*p <* 0.001), education (*p =* 0.002), marital status (*p =* 0.018), and diabetes prevalence (*p <* 0.001). The AD group was older, less educated, and had higher metabolic risk. Cognitive and functional measures (CDR-SB, MMSE, FAQ, NPI, ADAS-Cog 11/13, RAVLT) showed a clear decline from CN to MCI and AD (*all p <* 0.001).

**Table 1:** Demographic data table.

Characteristic	CN	MCI	AD	p. overall	p. CN vs. MCI	p. CN vs. AD	p. MCI vs. AD
	*N* = 96	*N* = 101	*N* = 37				
Age (years)	68.05 [65.27; 73.00]	72.90 [68.00; 76.50]	75.60 [71.20; 79.40]	<0.001	0.001	<0.001	0.037
Gender: Female (*n*)	41 (42.71%)	54 (53.47%)	24 (64.86%)	0.057	0.257	0.108	0.316
Education (years)	17.00 [16.00; 18.00]	16.00 [14.00; 18.00]	16.00 [13.00; 17.00]	0.002	0.014	0.004	0.15
Marital Status:Married	60 (63.16%)	76 (75.25%)	32 (86.49%)	0.018	0.139	0.048	0.236
Race: White	70 (73.68%)	84 (83.17%)	29 (78.38%)	0.555	0.738	0.845	0.845
Hypertension: Yes	46 (47.92%)	50 (49.50%)	16 (43.24%)	0.808	0.936	0.936	0.936
Diabetes: Yes	41 (42.71%)	18 (17.82%)	8 (21.62%)	<0.001	0.001	0.059	0.795
CDRSB	0.00 [0.00; 0.00]	1.00 [0.50; 2.00]	4.50 [3.50; 5.00]	<0.001	<0.001	<0.001	<0.001
MMSE	29.00 [28.00; 30.00]	28.00 [26.00; 29.00]	23.00 [21.00; 24.00]	<0.001	<0.001	<0.001	<0.001
FAQ	0.00 [0.00; 0.00]	2.00 [0.00; 5.00]	15.00 [11.00; 20.00]	<0.001	<0.001	<0.001	<0.001
MOCA	15.00 [12.00; 18.00]	22.00 [21.00; 24.00]	17.00 [13.00; 19.00]	<0.001	<0.001	0.231	<0.001
NPI	0.00 [0.00; 0.00]	1.00 [0.00; 6.00]	10.00 [5.00; 18.00]	<0.001	<0.001	<0.001	<0.001
GDS	1.00 [0.00; 2.00]	2.00 [1.00; 3.00]	2.00 [1.00; 3.00]	<0.001	<0.001	<0.001	0.916
ADAS11	4.67 [3.33; 7.00]	8.33 [6.67; 12.67]	19.67 [14.00; 22.33]	<0.001	<0.001	<0.001	<0.001
ADAS13	7.33 [4.67; 10.67]	14.33 [11.33; 21.00]	29.67 [24.67; 34.67]	<0.001	<0.001	<0.001	<0.001
RAVLT_immediate	46.50 [36.75; 55.00]	34.00 [29.00; 42.00]	23.00 [19.00; 26.00]	<0.001	<0.001	<0.001	<0.001
RAVLT_learning	6.00 [4.00; 8.00]	4.00 [2.00; 6.00]	2.00 [1.00; 3.00]	<0.001	<0.001	<0.001	<0.001
RAVLT_forgetting	4.00 [2.00; 6.00]	5.00 [3.00; 7.00]	5.00 [3.00; 6.00]	0.008	0.009	0.084	0.586
RAVLT_perc_forgetting	32.05 [13.33; 59.13]	57.14 [41.67; 87.50]	100.00 [100.00; 100.00]	<0.001	<0.001	<0.001	<0.001

### Functional imaging

**Figure 1:**
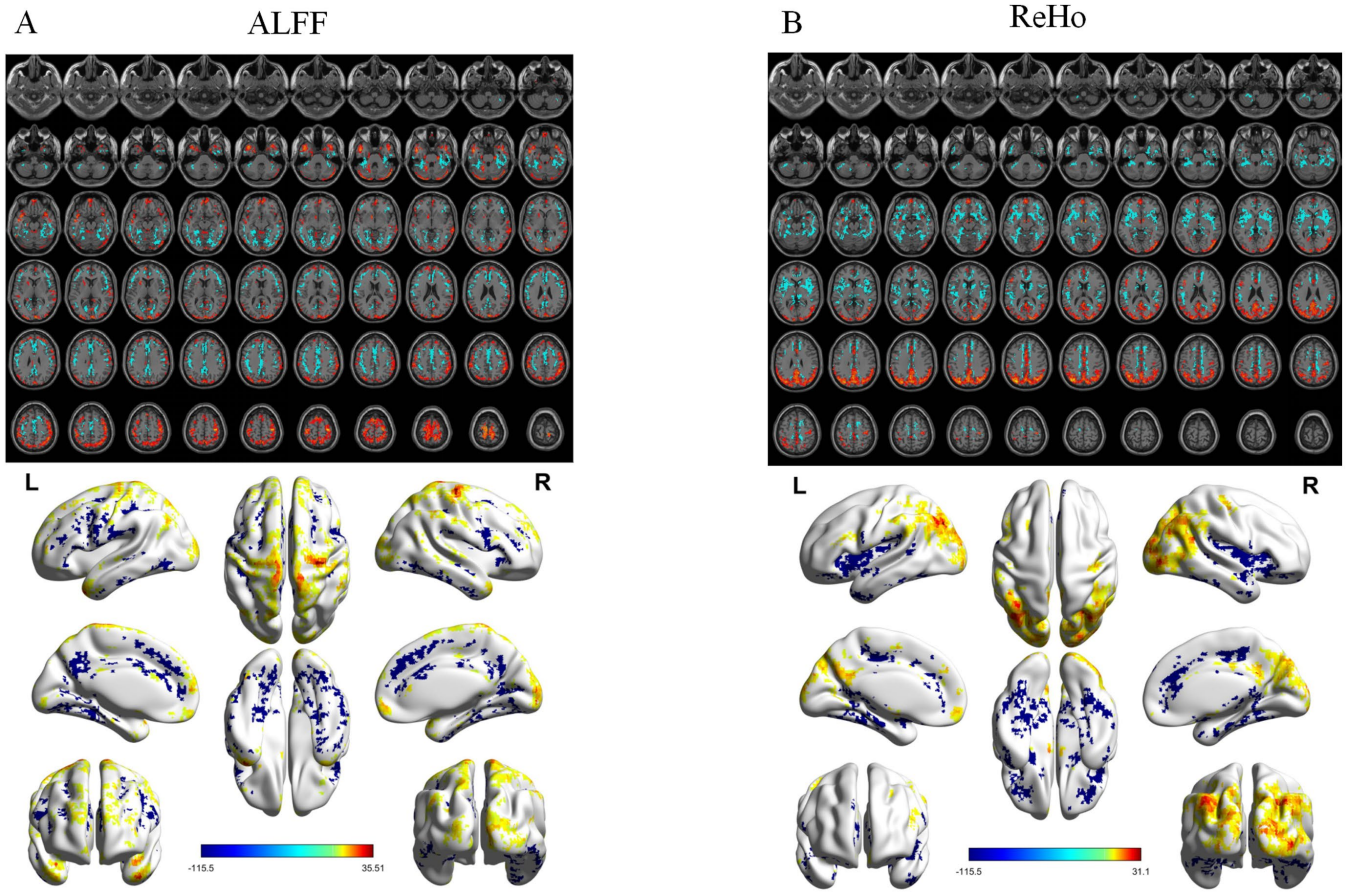
ANOVA analysis results of functional indices. **(A)** ALFF analysis results. **(B)** ReHo analysis results. From top to bottom: 2D planar visualization results, followed by 3D visualization results.

**Figure 2:**
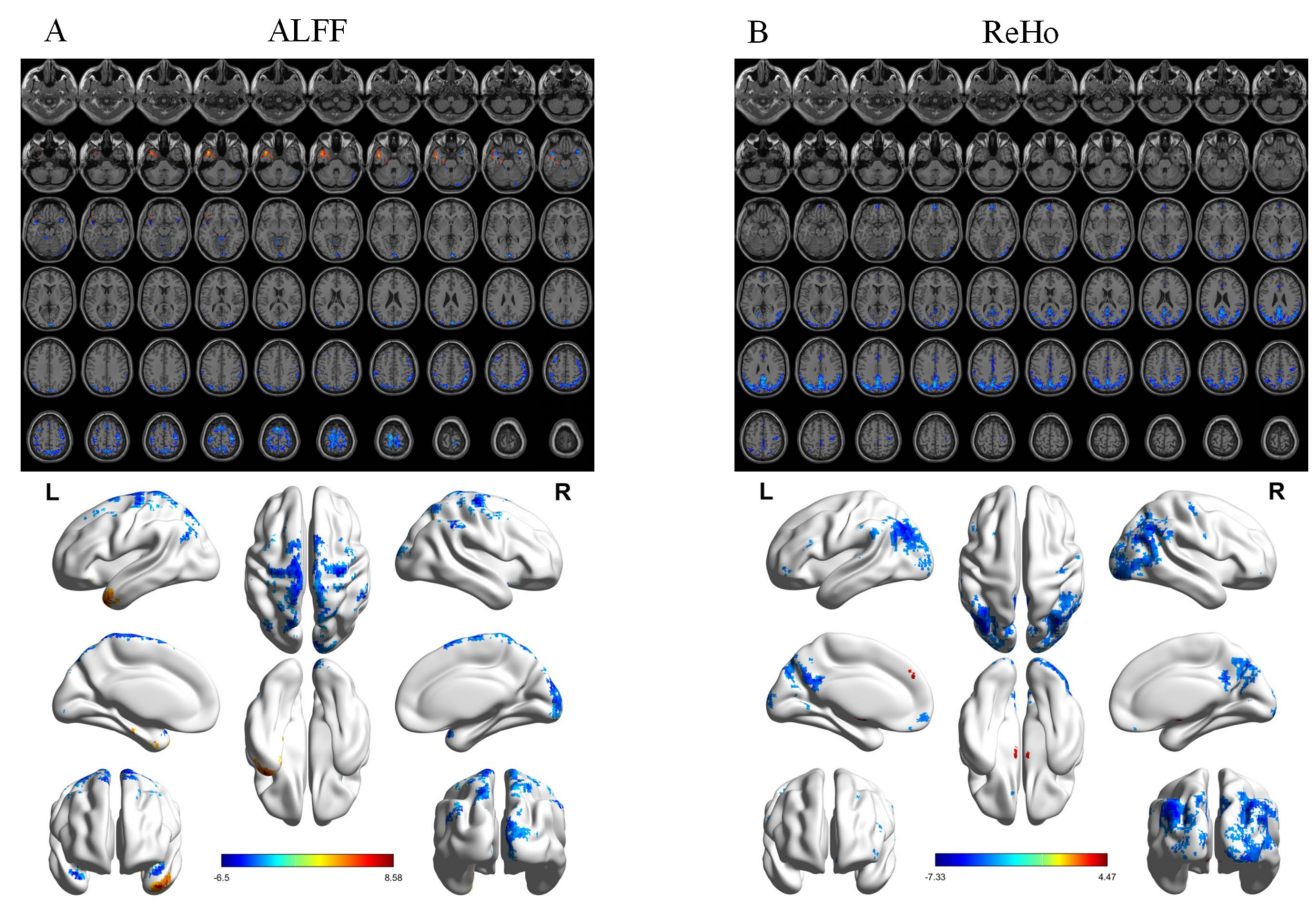
Post-hoc analysis results of functional indices for AD vs. CN. **(A)** ALFF analysis results. **(B)** ReHo analysis results. From top to bottom: 2D planar visualization results, followed by 3D visualization results.

**Figure 3:**
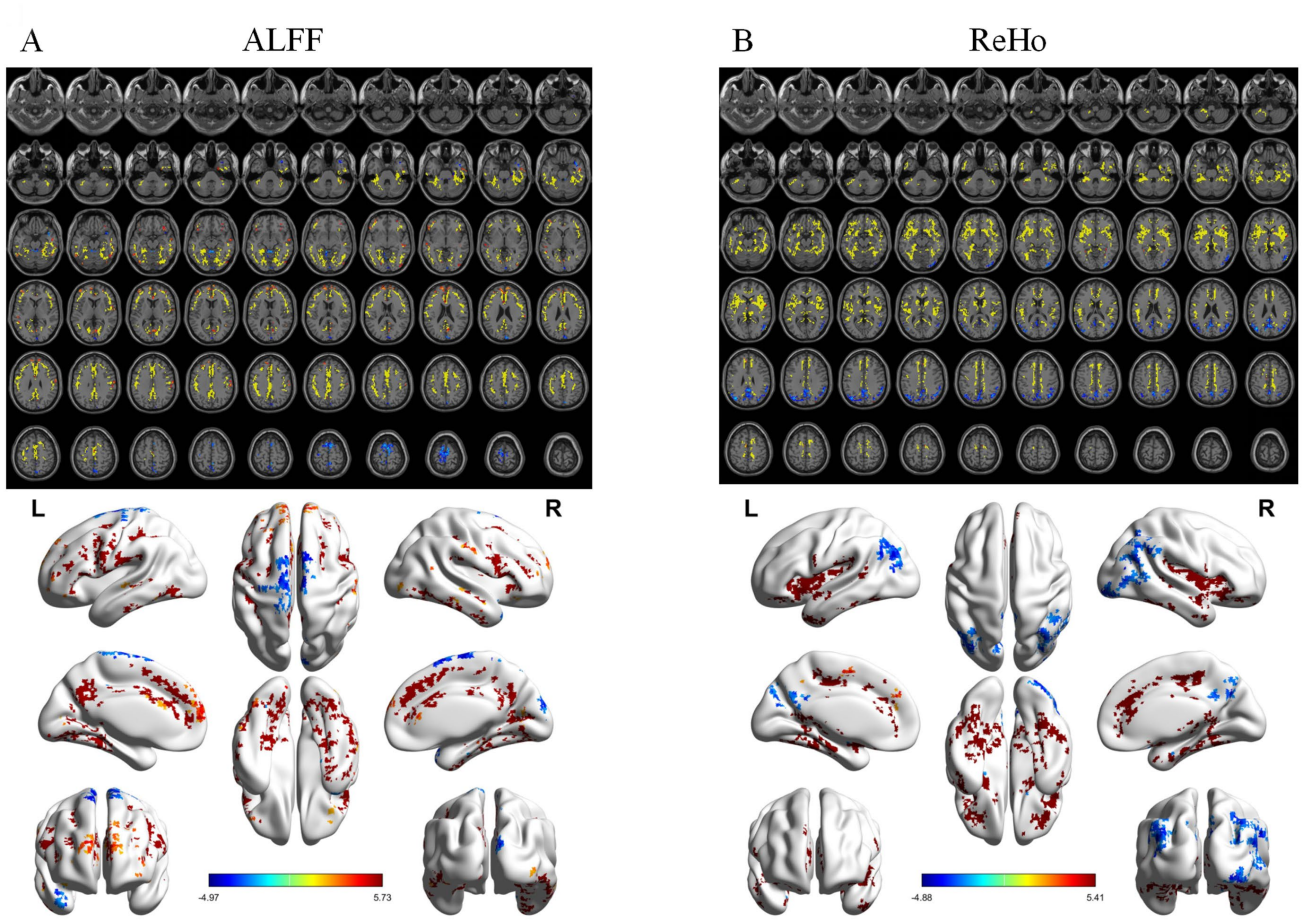
Post-hoc analysis results of functional indices for AD vs. MCI. **(A)** ALFF analysis results. **(B)** fALFF analysis results. C: ReHo analysis results. From top to bottom: 2D planar visualization results, followed by 3D visualization results.

**Figure 4:**
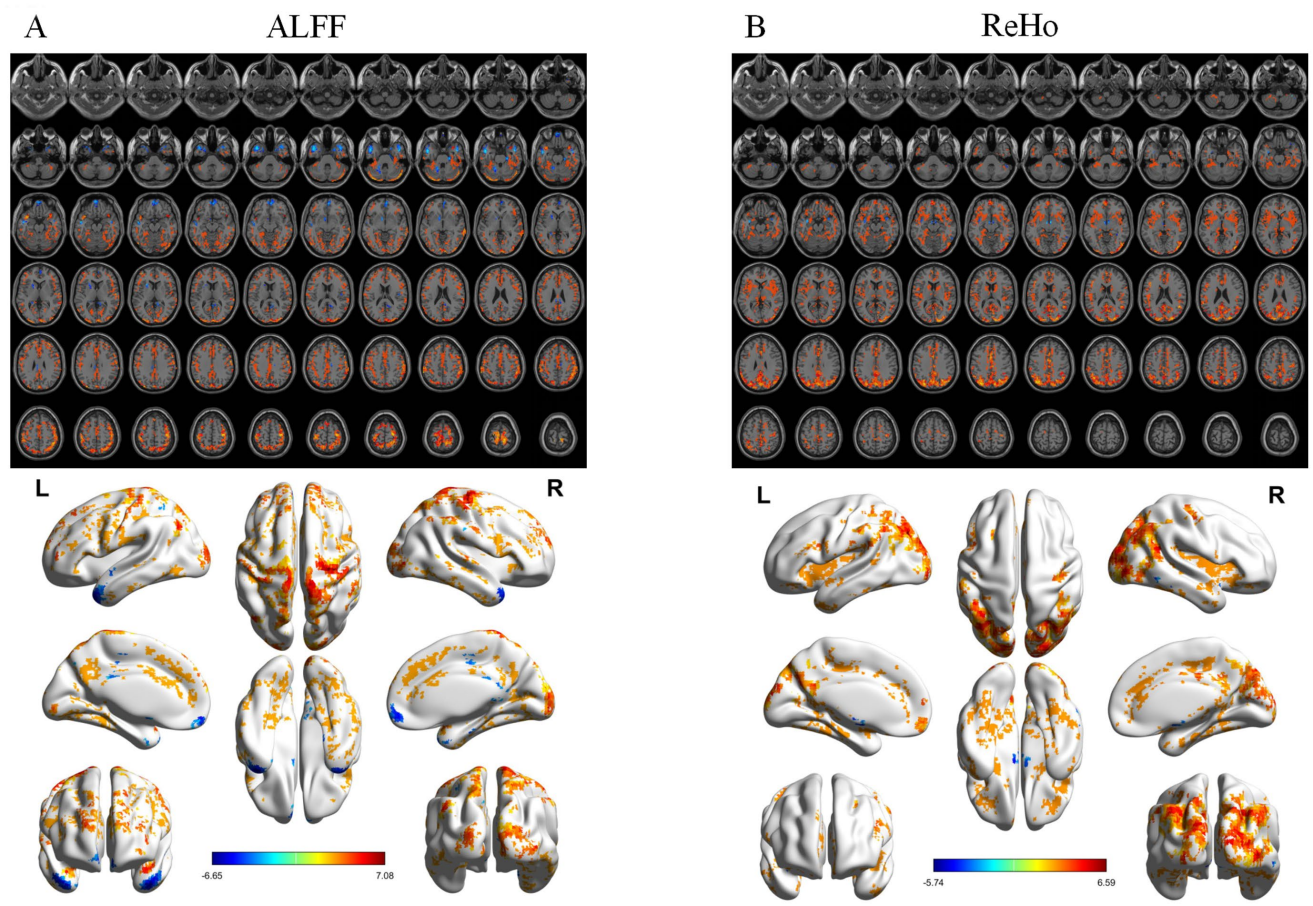
Post-hoc analysis results of functional indices for CN vs. MCI. **(A)** ALFF analysis results. **(B)** ReHo analysis results. From top to bottom: 2D planar visualization results, followed by 3D visualization results.

ALFF differed significantly among groups (Figure 1A), mainly in the right postcentral gyrus, right medial orbitofrontal cortex, and left putamen. *Post hoc* analyses showed progressive reductions from CN to MCI and AD (Figures 2–4). Compared with CN, AD exhibited reduced ALFF and ReHo in the right postcentral gyrus, left inferior temporal gyrus, right superior temporal pole, left middle frontal gyrus, and left cerebellar lobules IV–V. Compared with MCI, further reductions appeared in bilateral postcentral gyri, right supplementary motor area, right cuneus, and right middle temporal pole. MCI versus CN showed early ALFF declines in postcentral, inferior temporal, medial orbitofrontal/rectus, cerebellar Crus I, middle temporal gyrus, and putamen. No fALFF differences were detected.

ReHo differences were widespread (Figure 1B), primarily in the left middle occipital gyrus, left medial orbitofrontal cortex, right precentral gyrus, and left inferior temporal gyrus. AD versus CN reductions were noted in the occipital, orbitofrontal, and precentral regions, while AD versus MCI decreases involved the right insula, left middle cingulate, right angular gyrus, right inferior occipital gyrus, and left inferior temporal gyrus. Early ReHo declines were detectable at the MCI stage.

### Structural imaging

**Figure 5:**
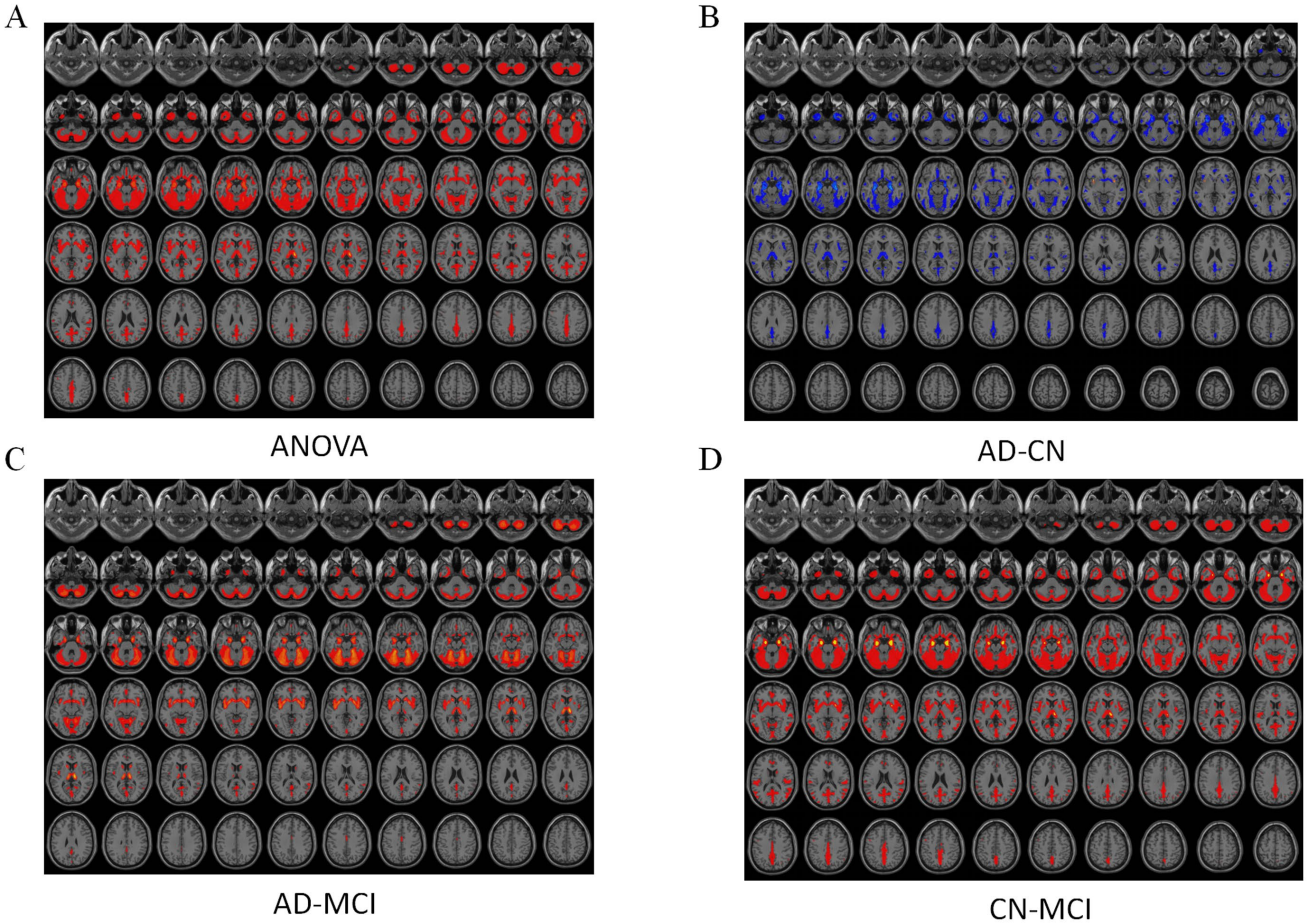
Statistical analysis results of gray matter volume. **(A)** ANOVA analysis results. **(B)** Post-hoc analysis results for AD vs. CN. **(C)** Post-hoc analysis results for AD vs. MCI. **(D)** Post-hoc analysis results for CN vs. MCI.

**Figure 6:**
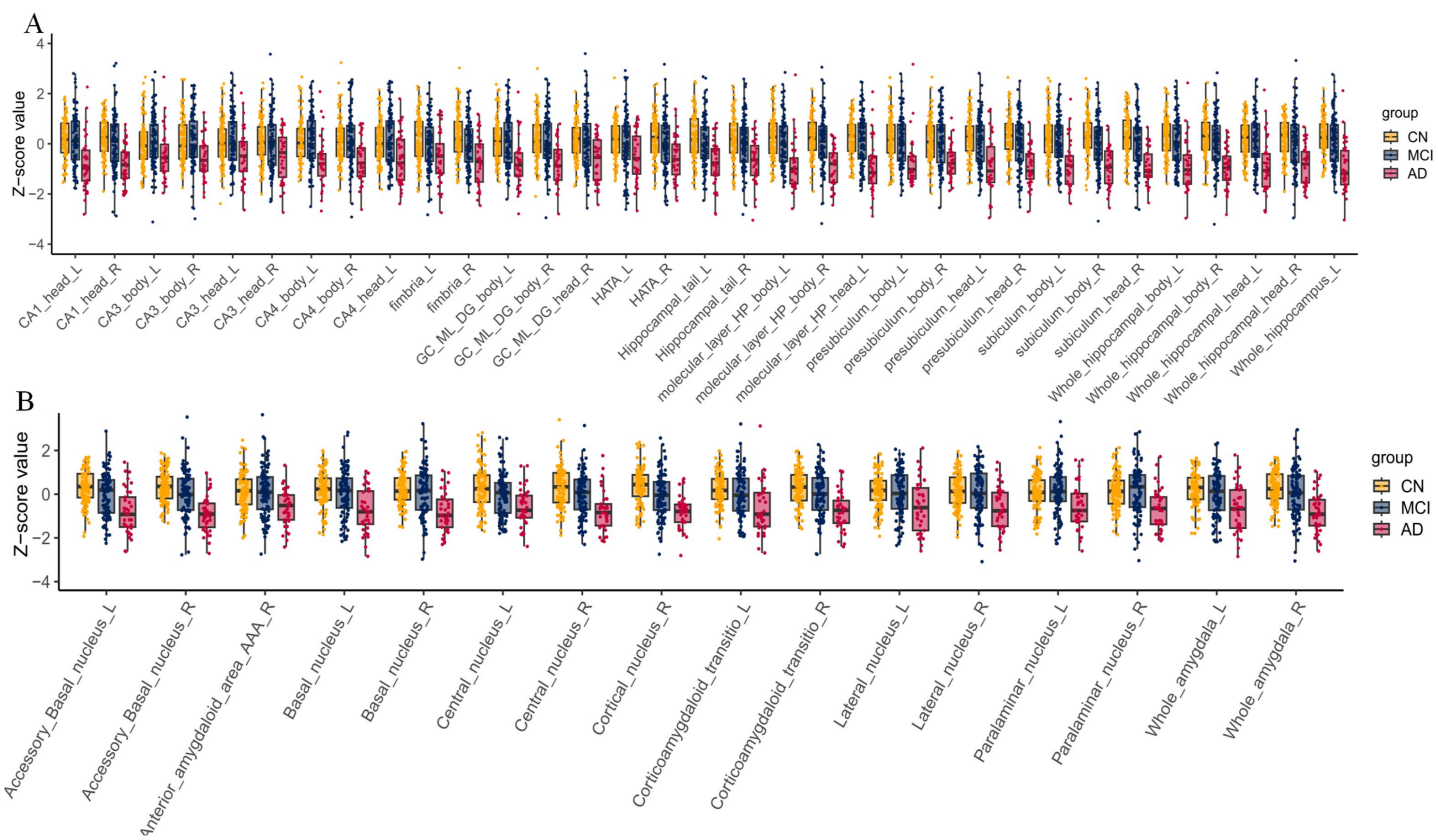
Analysis results of structural indices: **(A)** Hippocampal subfield analysis results and **(B)** amygdalar subfield analysis results. *p* < 0.05, FDR corrected.

Grey matter and hippocampal/amygdala subfield volumes differed significantly among groups (Figure 5; Table 2). AD showed widespread atrophy in the right hippocampus, bilateral thalami, right middle temporal gyrus, and right cerebellar lobules IV–V compared with CN and MCI. MCI displayed early volume reductions in the left para-hippocampal gyrus, right thalamus, and right middle occipital gyrus. Amygdala subfields showed stepwise atrophy from CN to MCI to AD, with prominent changes in the right basal, accessory basal, and cortical nuclei (Figure 6B). Hippocampal subfields showed similar patterns; AD exhibited widespread loss, while MCI changes were localised mainly to the right subiculum body (Figure 6A).

**Table 2:** Statistical analysis results of gray matter volume.

ANOVA	Brain region	MNI center of mass			F/T values	Cluster size (voxels)
		X	Y	Z		
	Hippocampus_R	28.5	−10.5	−19.5	2108.03	602
	Thalamus_R	10.5	−19.5	7.5	3404.79	1,064
	Occipital_Mid_R	33	−82.5	19.5	22.2768	168
	SupraMarginal_L	−61.5	−39	27	8.81484	66
	Frontal_Mid_Orb_L	−27	37.5	−16.5	17.9251	50
	Occipital_Mid_L	−28.5	−85.5	21	9.88879	66
	Frontal_Mid_Orb_R	35.5	34.5	−18	15.7188	30
	Precentral_L	−45	6	45	10.6686	50
AD-CN	Hippocampus_R	28.5	−10.5	−19.5	−32.894	548
	Thalamus_R Thalamus_L	7.5	−15	13.5	−8.83805	915
	Temporal_Mid_R	58.5	−37.5	4.5	−4.16903	743
	Insula_L	−37.5	18	1.5	−5.84966	533
	Cerebelum_8_R	18	−58.5	−51	−3.16342	109
AD-MCI	Cerebelum_4_5_R	21	−42	−21	33.5869	1,317
	Thalamus_R	10.5	−19.5	7.5	41.7359	831
	Cingulum_Ant_L	−6	49.5	−1.5	4.3892	273
	Temporal_Pole_Sup_R	48	16.5	−21	6.07168	224
	Temporal_Inf_L	−51	−10.5	−40.5	3.94367	152
	Temporal_Mid_R	57	−58.5	13.5	4.3892	145
	Cingulum_Mid_L	0	−4.5	37.5	3.47347	71
CN-MCI	ParaHippocampal_L	−21	−4.5	−31.5	1.17103	1,216
	Thalamus_R	7.5	−19.5	4.5	1.17103	1,064
	Occipital_Mid_R	33	−82.5	19.5	6.25464	168
	SupraMarginal_L	−61.5	−39	27	3.59693	66
	Frontal_Mid_Orb_L	−27	37.5	−16.5	5.12924	50
	Occipital_Mid_L	−28.5	−85.5	21	4.13144	66
	Precentral_L	−45	6	45	3.9571	50

### Diffusion imaging

Left and whole-brain DTI-ALPS indices differed among groups, while right DTI-ALPS did not (*p =* 0.173, FDR corrected). AD showed lower left and whole-brain indices than CN and MCI; CN and MCI did not differ. Hippocampal subregional diffusion metrics were largely stable, but RD and MD in right subregion X215 and MD in X226R increased at MCI and AD stages (Table 3).

**Table 3:** Statistical analysis results of DTI indicators for three study groups (mean ± SD).

DTI	CN (*n* = 96)	MCI (*n* = 101)	AD (*n* = 37)	*F*	*p*
hippRD_X215 RD( × 10⁻^3^mm^2^/s)	0.85 ± 0.06	0.92 ± 0.08	1.05 ± 0.10	19.86	<0.001
hippRD_X215 MD( × 10⁻^3^mm^2^/s)	1.02 ± 0.07	1.09 ± 0.09	1.21 ± 0.11	16.52	<0.001
hippMD_X226R MD( × 10⁻^3^mm^2^/s)	1.05 ± 0.08	1.12 ± 0.09	1.23 ± 0.12	12.36	<0.001
ALPS_L	1.89 ± 0.13	1.81 ± 0.16	1.58 ± 0.19	22.56	<0.001
ALPS_R	1.82 ± 0.14	1.79 ± 0.15	1.72 ± 0.17	2.15	0.173
ALPS	1.85 ± 0.12	1.78 ± 0.15	1.62 ± 0.18	18.95	<0.001

*p < 0.05, FDR corrected.

### Correlation analysis

**Figure 7:**
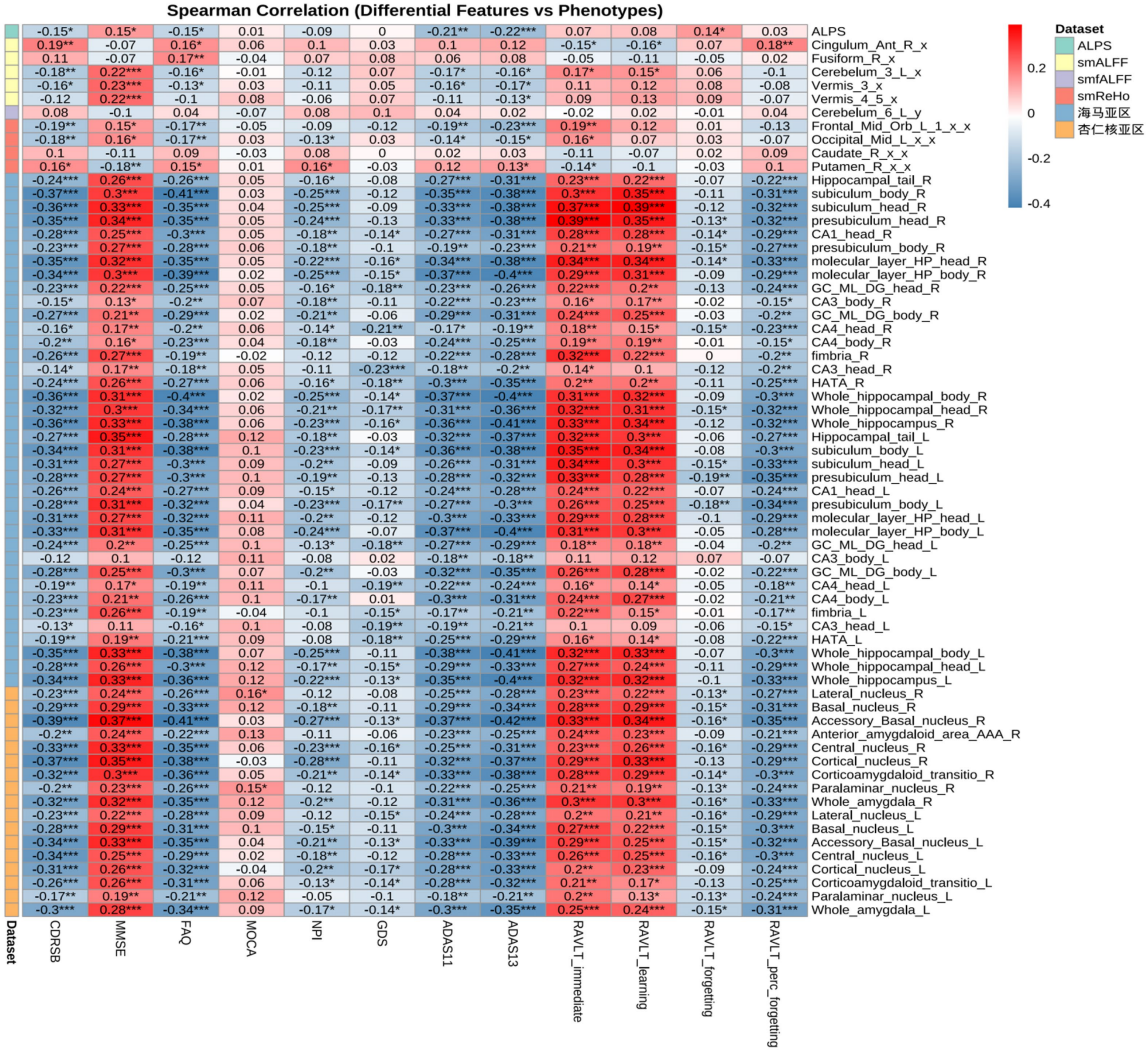
Correlation analysis between differential features (overall ANOVA, Tukey: AD-CN and MCI-AD differences) and phenotypes. *p* < 0.05, FDR corrected.

**Figure 8:**
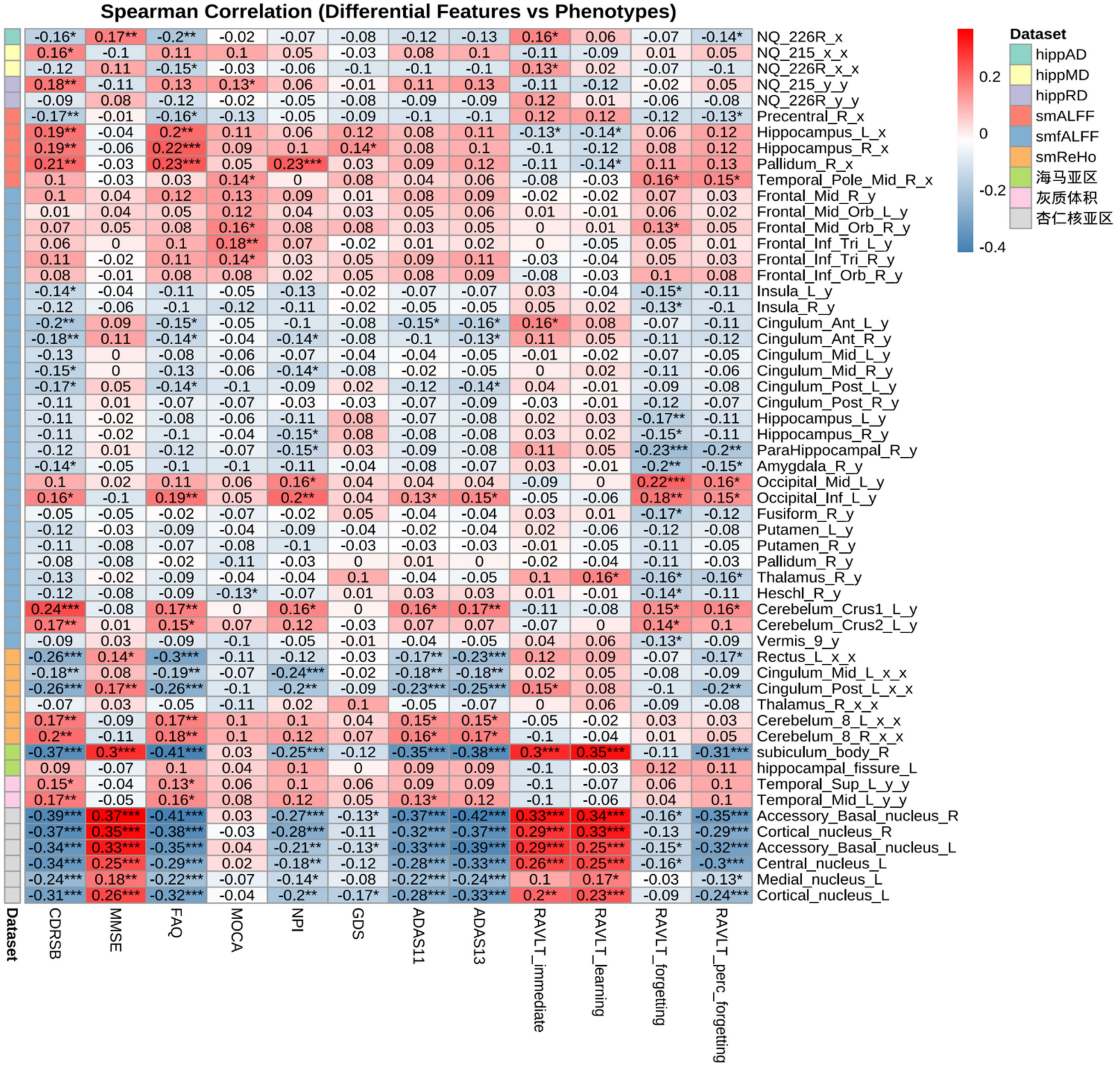
Correlation analysis between differential features (overall ANOVA, Tukey: MCI-CN differences) and phenotypes.

Spearman correlations (Figures 7, 8) revealed modality-specific associations with cognition. Frontoparietal regions (middle frontal, middle cingulate, angular gyrus) correlated with MMSE and ADAS-Cog, temporal regions with NPI and FAQ (r = 0.16–0.18), and limbic/associative regions (para-hippocampal gyrus, left inferior frontal triangular, insula) with RAVLT memory scores.

### Machine learning

**Figure 9:**
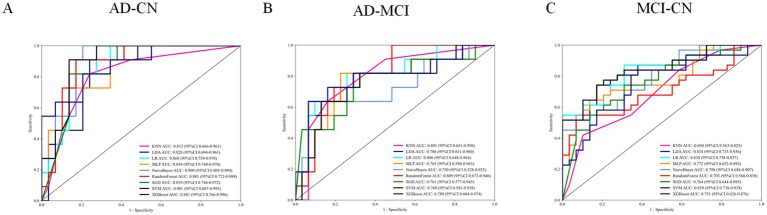
Interpretability analysis of ROC curves. **(A)** AD-CN ROC curve. **(B)** AD-MCI ROC curve. **(C)** MCI-CN ROC curve.

The multimodal ML framework achieved robust classification (Figure 9). AD vs. CN: SVM and Naive Bayes AUCs = 0.901(95% CI: 0.807–0.995) and 0.900 (95% CI: 0.806–0.994); SVM accuracy = 0.850. AD vs. MCI: RF AUC = 0.809 (95% CI: 0.673–0.946). MCI vs. CN: SVM AUC = 0.839 (95% CI: 0.738–0.939) and LR AUC = 0.838 (95% CI: 0.738–0.937); accuracy = 0.767. All ROC curves were significant (*p <* 0.05; Tables 4–6).

**Table 4:** Machine learning classification results for AD vs. CN.

Model	AUC	AUC 95% CI	Acc	Sen	Spe
LR	0.868	0.7591–0.9775	0.725	0.909	0.655
NaiveBayes	0.900	0.8055–0.9939	0.825	0.909	0.793
KNN	0.813	0.6663–0.9606	0.775	0.455	0.897
RandomForest	0.881	0.7723–0.9894	0.825	0.818	0.828
XGBoost	0.881	0.7656–0.9962	0.775	0.727	0.793
MLP	0.859	0.7398–0.9781	0.675	0.909	0.586
SVM	0.901	0.8073–0.9952	0.850	0.818	0.862
LDA	0.828	0.6945–0.9607	0.775	0.818	0.759
SGD	0.859	0.7459–0.9720	0.825	0.727	0.862

**Table 5:** Machine learning classification results for AD vs. MCI.

Model	AUC	AUC 95% CI	Acc	Sen	Spe
LR	0.806	0.6484–0.9645	0.738	0.727	0.742
NaiveBayes	0.730	0.5283–0.9321	0.833	0.545	0.935
KNN	0.801	0.6510–0.9502	0.810	0.455	0.935
RandomForest	0.809	0.6731–0.9457	0.762	0.636	0.806
XGBoost	0.789	0.6039–0.9738	0.833	0.545	0.935
MLP	0.765	0.5880–0.9428	0.762	0.727	0.774
SVM	0.760	0.5810–0.9380	0.786	0.545	0.871
LDA	0.786	0.6115–0.9604	0.714	0.727	0.710
SGD	0.761	0.5770–0.9450	0.690	0.727	0.677

**Table 6:** Machine learning classification results for CN vs. MCI.

Model	AUC	AUC 95% CI	Acc	Sen	Spe
LR	0.838	0.7378–0.9374	0.767	0.839	0.690
NaiveBayes	0.798	0.6880–0.9072	0.717	0.516	0.931
KNN	0.694	0.5632–0.8250	0.517	0.097	0.966
RandomForest	0.703	0.5678–0.8382	0.700	0.484	0.931
XGBoost	0.751	0.6258–0.8759	0.717	0.774	0.655
MLP	0.772	0.6520–0.8920	0.733	0.613	0.862
SVM	0.839	0.7381–0.9393	0.767	0.710	0.828
LDA	0.834	0.7328–0.9357	0.767	0.710	0.828
SGD	0.764	0.6436–0.8848	0.700	0.710	0.690

**Figure 10:**
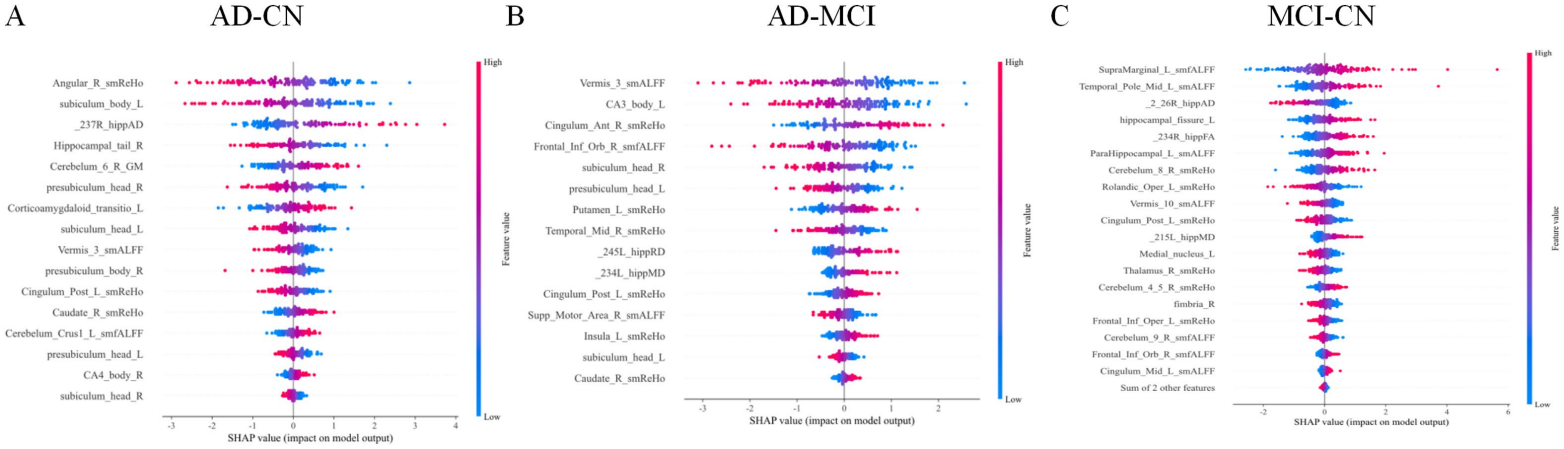
SHAP interpretability analysis. **(A)** AD-CN interpretability analysis. **(B)** AD-MCI interpretability analysis. **(C)** MCI-CN interpretability analysis.

SHAP analysis identified key features (Figure 10). AD-CN classification relied on right angular gyrus ReHo and left hippocampal subiculum body integrity. AD-MCI distinctions involved the cerebellar vermis, hippocampal CA3, and anterior cingulate cortex. MCI-CN differences depended on left supramarginal and middle temporal activity and early hippocampal fissure microstructural changes. Feature importance progressed from limbic to cortical regions, illustrating interpretable neuropathological evolution across the AD spectrum.

## Discussion

Multi-index rs-fMRI analyses (ALFF, fALFF, ReHo) revealed widespread changes in spontaneous neural activity and local synchronization across the AD spectrum. Early ALFF alterations in the right postcentral gyrus and medial orbitofrontal cortex align with prior findings of sensorimotor and executive network impairment (Tang et al., 2024). ReHo analyses showed disrupted synchrony within the DMN (e.g., left middle occipital gyrus) and SN (e.g., right insula), indicating that AD pathology involves large-scale network dysfunction rather than isolated regional damage (Cai et al., 2018). Notably, abnormal cerebellar–cortical circuit activity (Crus I, lobules IV–V) was detected at the MCI stage, supporting the cerebellum’s role in higher-order cognition (Yuan et al., 2022; Lyu et al., 2021; Zhou et al., 2021; Li et al., 2023).

Structural analyses confirmed progressive brain atrophy along the AD continuum. AD patients exhibited marked grey matter reductions in the right hippocampus and left precuneus, closely linked to cognitive decline, with hippocampal subregions showing particularly pronounced loss (Zilioli et al., 2024; Pusparani et al., 2025). Amygdala subregion atrophy emerged between MCI and CN, suggesting early warning potential (Shahid et al., 2022). Temporal and precuneus volume loss was evident at the AD stage, consistent with pathology spreading from medial temporal to association cortices (Rao et al., 2022). Elevated RD and MD in hippocampal subregions at MCI indicate microstructural damage preceding macroscopic atrophy, highlighting their value for early diagnosis (Zhang et al., 2020; Zhao et al., 2019).

Diffusion imaging and DTI-ALPS indices clarified microstructural and clearance-pathway mechanisms. AD and MCI showed reduced left and whole-brain DTI-ALPS indices, reflecting impaired glymphatic clearance and lateralized vulnerability (Huang et al., 2024; Steward et al., 2021; Taoka et al., 2024). Elevated RD in right hippocampal subregion X215 in MCI indicated early myelin damage, while localized abnormalities in X215 and X226R suggest regional white matter compromise linked to cognitive decline (Khatri and Kwon, 2022; Zhang et al., 2025; Zhou et al., 2022; Ahmadzadeh et al., 2025).

Multimodal machine-learning analyses showed moderate-to-good discrimination among diagnostic groups based on integrated neuroimaging features. Frontal and temporal functional abnormalities were associated with cognitive and behavioral scores (ADAS11/13, MMSE, RAVLT, NPI, and FAQ), while structural changes in the parahippocampal region were related to memory performance. Among the evaluated models, SVM achieved the highest AUCs for AD-CN and MCI-CN classification (AUC = 0.901 and 0.839, respectively), and SHAP analyses identified influential features including right angular gyrus ReHo and left hippocampal subiculum-related measures. These findings suggest that multimodal imaging combined with machine learning may help capture clinically relevant heterogeneity across the AD spectrum. However, SHAP values should be interpreted as measures of model-level feature contribution rather than as evidence of causal biological effects. In addition, although the analytical pipeline was designed to reduce information leakage by separating training and held-out test data, the relatively limited sample size warrants cautious interpretation of classification performance (Raza et al., 2025; Odusami et al., 2024).

Several limitations should be acknowledged. First, this was a retrospective cross-sectional study, and therefore the observed associations should not be interpreted as evidence of temporal progression or causality. Second, although demographic variables were considered as potential confounders in the statistical analyses, a formal ablation analysis comparing demographic-only, imaging-only, and combined models was not performed; thus, the incremental predictive contribution of demographic variables remains to be determined. Third, the sample size was relatively limited, particularly in the AD group (*n* = 37), which may increase the risk of optimistic bias and reduce the stability of model estimates despite the use of a held-out test set. Finally, all analyses were conducted using ADNI data, and independent external validation will be necessary before broader clinical generalization. Future studies should expand sample size, include more AD cases, incorporate external datasets, and evaluate multimodal imaging together with PET, cerebrospinal fluid, or other biomarker modalities.

## Conclusion

Multimodal neuroimaging revealed functional, structural, and microstructural alterations across AD, MCI, and CN groups. Integrated rs-fMRI, sMRI, and DTI analyses provided insights into the neurobiological basis of cognitive decline. Correlation and ML analyses identified potential biomarkers and auxiliary tools for early AD diagnosis, supporting clinical translation and early intervention strategies.

## Data Availability

The MRI data analyzed in this study were obtained from the Alzheimer’s Disease Neuroimaging Initiative (ADNI) database (https://adni.loni.usc.edu). ADNI data are publicly available upon qualified request. The derived features and analysis code are included in the supplementary material. Further inquiries can be directed to the corresponding author.

## Footnotes

1
http://adni.loni.usc.edu/

2
http://fmri.org/DPARSF

3
http://www.fil.ion.ucl.ac.uk/spm

4
https://github.com/Jfortin1/ComBat-Harmonization
